# MARITAL CLOSENESS, SPOUSAL SIMILARITY, AND COGNITION: THE GENDERED IMPACT OF DISCORDANT PERCEPTIONS

**DOI:** 10.1093/geroni/igad104.0898

**Published:** 2023-12-21

**Authors:** Yue Qin, Sara Moorman, Michal Engelman

**Affiliations:** University of Wisconsin-Madison, Madison, Wisconsin, United States; Boston College, Boston, Massachusetts, United States; University of Wisconsin-Madison, Madison, Wisconsin, United States

## Abstract

Many older adults are in long-term marriages, and an emerging literature examines the impact of marital attributes on later-life cognitive function. While both spouses’ perceptions of the marital relationship may influence health outcomes in the couple, most research only captures one partner’s report. Using unique data on 2,791 heterosexual married couples from the Wisconsin Longitudinal Study, we analyze the dyad’s evaluations of their similarity and closeness in 2004 and the impact of concordance or discordance in these marital attributes on cognitive health in 2020, when participants were approximately 80 years old and cognitive variation is more manifest. Results from OLS regressions reveal notable gender differences. Compared with dyads in which both spouses reported being “very similar,” women in marriages with discordant views on the partners’ similarity had significantly lower cognition in 2020 (wife gave the poorer rating: b=-0.74, p<.05; husband gave the poorer rating: b=-0.84, p<.05). Men’s cognitive function was not influenced by assessments of similarity in the couple. Furthermore, compared with dyads in which both spouses reported being “very close,” men in marriages with discordant views on the partners’ closeness had significantly lower cognition in 2020 (woman gave the poorer rating: b= -0.88, p < .05). Women’s cognitive function was unrelated to dyadic reports of closeness. Our results suggest that within-couple discordance in assessment of marital closeness and spousal similarity – more than marital attributes themselves – influences later-life cognitive function for women and men in varied ways.

